# Genome-Wide Association Studies on the Autosomes and Chromosome X Uncover Genetic Basis of Reproductive Traits in Yorkshire Pigs

**DOI:** 10.3390/ani16050750

**Published:** 2026-02-27

**Authors:** Teddy Tinashe Chitotombe, Qing Lin, Wondossen Ayalew, Zhe Zhang

**Affiliations:** State Key Laboratory of Swine and Poultry Breeding Industry, Guangdong Provincial Key Lab of Agro-Animal Genomics and Molecular Breeding, College of Animal Science, South China Agricultural University, Guangzhou 510642, China; tedchitoz@gmail.com (T.T.C.); qing_lin1996@126.com (Q.L.); wondessenayalew9@gmail.com (W.A.)

**Keywords:** Yorkshire pigs, autosomes, X chromosome, reproductive trait, GWAS

## Abstract

Reproductive efficiency is a fundamental determinant of productivity in pig breeding programs. However, the role of the X chromosome in shaping the genetic basis of reproductive traits remains underexplored. To address this problem, Genome-Wide Association Studies were conducted to explore the genetic basis of reproductive traits in Yorkshire pigs. We collected data from 2659 Yorkshire sows, focusing on four reproductive traits across the first three parties. Statistical analyses included principal component analysis, estimation of genetic parameters, and mixed linear model association to identify SNPs and candidate genes. Heritability estimates (*h*^2^) ranged from 0.037 (TLWT_BA_P1) to 0.215 (TNB_P3). Genome-wide association analysis identified 23 significant SNPs and candidate genes, including several putatively significant X-linked genes; however, only a single X-linked locus was significantly associated with one trait. These findings demonstrate that, for 9 of the 12 parities, the proportion of total heritability attributable to X-linked variation was 0–2.7%. For the remaining three parities, the corresponding proportions were 8.4%, 21.5%, and 50.9%, while autosomes accounted for most of the genetic variation. In all but one trait, heritability estimated from X-linked GRMs was not significantly different from zero, highlighting the importance of incorporating X-linked information into genomic selection strategies for improving reproductive efficiency in pigs.

## Simple Summary

Understanding the genetic basis influencing reproductive performance in pigs is essential for improving breeding efficiency and productivity. In this study, we examined the genetic basis of key reproductive traits in Yorkshire pigs by analyzing genetic information from both autosomes and the X chromosome. We identified genetic regions and candidate genes associated with total litter weight of piglets born alive (TLWT_BA), total number born (TNB), total litter weight at 21 days (TLWT_D21), and number of weaned pigs (NWEAN). Importantly, the inclusion of the X chromosome revealed additional genetic effects that are often overlooked in studies focusing solely on autosomes. These findings provide new insights into the genetic determinants of reproduction in pigs and offer useful genetic markers that may support the development of more effective breeding strategies to enhance reproductive efficiency in Yorkshire populations.

## 1. Introduction

Pigs are among the earliest domesticated animals, and selective breeding has produced diverse breeds tailored to consumer demands [1]. The Yorkshire pigs play a central role in global pork production due to their superior reproductive performance, adaptability, and widespread use in crossbreeding programs [2]. Reproductive traits such as total number born (TNB), number born alive (NBA), and gestation length (GL) are key determinants of productivity and profitability [3,4,5]. Understanding the genetic basis of these traits is, therefore, critical for optimizing breeding strategies and improving herd performance. The prominence and genetic significance of Yorkshire pigs make them an ideal model for investigating the genetic basis of pig reproduction [6].

Genome-wide association studies have revolutionized animal genetics by enabling the analysis of associations between single-nucleotide polymorphisms (SNPs) and growth traits [7,8], feed efficiency [9], meat quality [10], semen traits [11,12], and complex traits [13]. Advances in high-density SNP genotyping and whole-genome sequencing have expanded the scope of GWAS in livestock, providing accurate evaluations and facilitating the identification of candidate genes underlying economically important traits [3,14]. This has positioned GWAS as an essential tool for accelerating genetic improvement programs in pigs [15].

Despite extensive progress, most GWAS in mammals, including cattle [16] and humans [17], have predominantly focused on autosomes, often overlooking the X chromosome due to its unique inheritance patterns [18], characterized by male hemizygosity across the non-pseudoautosomal region [19,20], and dosage compensation [21]. Despite these complexities, the Genome-wide Complex Trait Analysis (GCTA v1.95) software offers a robust and adequate single-model approach for analyzing the inheritance patterns of the X chromosome [22], particularly in female-only populations, where dosage and sex-specific effects are inherently controlled [23]. Nonetheless, growing evidence indicates that the X chromosome contributes substantially to milk yield [24], male fertility [25], and body size [26]. Therefore, a comprehensive genetic analysis requires the inclusion of both autosomal and X-linked markers to fully elucidate the genetic basis underlying reproductive performance in pigs [26].

In this study, we employed GWAS to identify SNPs on autosomes and the X chromosomes associated with four reproductive traits in Yorkshire pigs, including TNB, NWEAN, TLWT_BA, and TLWT_D21. Gene Ontology (GO) enrichment analysis, Kyoto Encyclopedia of Genes and Genomes (KEGG) pathway analysis, and functional annotation of candidate genes were conducted to better understand the functional roles and biological pathways of the candidate genes in the organism. This study’s outcomes provide valuable insights that pave the way for future genetic improvement of sows with efficient reproduction performances, thereby enhancing sow fertility and productivity within contemporary pig breeding programs.

## 2. Materials and Methods

### 2.1. Phenotype and Genotype Data Quality Control

The study population consisted of Yorkshire pigs raised on a farm managed by China Guangxi State Farms Yongxin Animal Husbandry Group Co., Ltd. (located in Nanning, Guangxi, China). All animals were maintained under uniform nutritional and management conditions. A total of 2659 sows with both phenotypic and genotypic records were included in the analysis; however, the exact number of observations available for each trait and parity varied.

Genotypes of 2659 sows were obtained using a 50 K SNP array and structured into three datasets: autosomal SNPs (47,788 markers), 4009 markers located in the non-pseudoautosomal region (non-PAR) of the X chromosome, and a combined set encompassing both (51,797 markers). Quality control of all datasets was conducted using PLINK v1.9 [27]. Individuals with call rates ≤90% (--mind 0.10), SNPs with call rates ≤90% (--geno 0.10), and minor allele frequency <5% (--maf 0.05) were excluded. After filtering, the autosomal, X chromosome, and combined datasets retained 39,048, 1,967, and 41,015 SNPs, respectively.

Phenotypic records from the first three parities, first (P1), second (P2), and third (P3), were included in this analysis. The traits analyzed were: (i) total litter weight of piglets born alive (TLWT_BA), defined as the sum of weights (kg) of all piglets born alive in a litter, measured immediately after farrowing; (ii) total number born (TNB), defined as the total count of piglets born per litter; (iii) total litter weight at 21 days (TLWT_D21), defined as the combined weight (kg) of all surviving piglets in a litter at 21 days of age; and (iv) number of weaned piglets (NWEAN), defined as the number of piglets per litter surviving with the sow or present in the assigned weaning group at weaning. Phenotypic data were summarized in R (version 4.4.1) using descriptive statistics, including mean, standard deviation, coefficient of variation, and minimum and maximum values for each trait by parity.

### 2.2. Genomic Relationship Matrices (GRM)

Three different genomic relationship matrices (GRMs) were then constructed using the SNPs that passed the quality control. The first matrix ($g_{A}$) was based on 39,048 autosomal SNPs, the second matrix ($g_{X}$) included 1967 SNPs from the X chromosome, and the third matrix ($g_{ALL})$ combined both sets, totaling 41,015 SNPs. Since all sampled animals were females, no adjustments were made regarding dosage compensation for the X chromosome (both X chromosomes are active in females). All matrices were built using the --*make-grm* function in the GCTA software [22], which was originally designed for autosomal SNPs.

### 2.3. Population Structure

Principal component analysis (PCA) was conducted separately on autosomes and X chromosomes to assess population structure and identify potential sources of genetic stratification in the Yorkshire population. Eigenvalues and eigenvectors were calculated using GCTA software based on filtered SNPs. The PCA results were visualized using the “ggplot2” package in R.

### 2.4. Estimation of Genetic Parameters

To estimate the relative proportions of genetic variance explained by the autosomes and the X chromosome, and the corresponding heritabilities, REML analyses were carried out separately on the autosomes and X chromosomes for each trait per parity using GCTA software [22].

The model used for autosomes is as follows:

$$y=Xb+g_{A}+e$$

The model used for X chromosomes is as follows:

$$y=Xb+g_{X}+e$$

where y is the vector of phenotypic values; b is the fixed effects, including year farrowing, season farrowing, and current farm; X is the design matrix for fixed effects b; $g_{A}\sim N(0,G_{A}\sigma_{A}^{2})$ is the vector of random autosomal genetic effects, with $g_{A}$ the autosomal GRM and $\sigma_{A}^{2}$ the autosomal genetic variance; $g_{X}\sim N(0,G_{X}\sigma_{X}^{2})$ is the vector of random X-linked genetic effects, with $g_{X}$ the X chromosome GRM and $\sigma_{X}^{2}$ the X-linked genetic variance; and $e\sim N(0,I\sigma_{e}^{2})$ is the vector of random residual effects, with I the identity matrix and $\sigma_{e}^{2}$ the residual variance. Then, we calculated the overall heritability of the traits $\left(h^{2}=(\sigma_{A}^{2}+\sigma_{X}^{2})/(\sigma_{A}^{2}+\sigma_{X}^{2}+\sigma_{e}^{2})\right)$ and the heritability due to autosomes $\left(h_{AUT}^{2}=\sigma_{A}^{2}/(\sigma_{A}^{2}+\sigma_{X}^{2}+\sigma_{e}^{2})\right)$ and the X chromosome $\left(h_{X}^{2}=\sigma_{X}^{2}/(\sigma_{A}^{2}+\sigma_{X}^{2}+\sigma_{e}^{2})\right)$.

### 2.5. Correlation Analysis

To evaluate the relationships among the four reproductive traits, we performed both phenotypic (Pearson’s) and genetic correlation analyses across the first three parities. Phenotypic correlations were calculated in R using the base stats function cor.test, with data handling performed via the “data.table” package. Genetic correlations were estimated using GCTA software. Both phenotypic and genetic correlations were computed in R (version 4.4.1), and visualization was performed using the “ggplot2” package.

### 2.6. Genome-Wide Association Studies (GWAS)

We performed genome-wide association analyses for each trait and parity using the mixed linear model association (MLMA) implemented in GCTA software [22]. To identify putative association signals, we implemented a suggestive significance threshold of $p<1\times 10^{-4}$. Within this framework, SNPs surpassing this threshold were prioritized as candidate loci, a strategy employed to enhance the reliability and robustness of our findings while minimizing the risk of excluding potentially biologically relevant variants [28,29]. Due to the small sample size in the X chromosome dataset, we used $g_{A}$ as the GRM for the X chromosome to avoid a significant loss of statistical power for the association analyses, consistent with approaches adopted in previous studies [24]. The association analysis model used is as follows:

$$y=Xb+x\beta +g_{A}+e$$

where *y* is the phenotype; b is the fixed effects, including year farrowing, season farrowing, and current farm; X is the design matrix for fixed effects b; x is the SNP genotype indicator variable coded as 0, 1, or 2; β is the additive effect (fixed effect) of the candidate SNP to be tested for association; $g_{A}\sim N(0,G_{A}\sigma_{A}^{2})$ is the vector of random autosomal genetic effects, with $g_{A}$ the autosomal GRM and $\;\sigma_{A}^{2}$ the autosomal genetic variance; and $e\sim N(0,I\sigma_{e}^{2})$ is the vector of random residual effects, with I the identity matrix and $\sigma_{e}^{2}$ the residual variance. After performing GCTA-MLMA, we conducted conditional analysis based on the GWAS summary statistics for each trait per parity using GCTA-COJO [30]. We utilized the corresponding genotype data as the LD reference panel.

### 2.7. Candidate Gene and Functional Annotation

Candidate genes associated with significant SNPs were defined as those located within 1 Mb upstream or downstream of each SNP, based on the pig reference genome (Sus scrofa 11.1) (https://www.ncbi.nlm.nih.gov/datasets/genome/GCF_000003025.6, accessed on 15 August 2025) using “GenomicRanges” and “rtracklayer” packages in R. Gene Ontology (GO) enrichment analysis and Kyoto Encyclopedia of Genes and Genomes (KEGG) pathway analysis were conducted using the annotations associated with significant SNPs. Enrichment analyses were performed using the “clusterProfiler” package in [31].

## 3. Results

### 3.1. Genetic and Phenotypic Data

After quality control, a total of 39,048 autosomal SNPs and 1967 X chromosome SNPs were retained for further marker analysis. The genome-wide distribution of these SNPs across autosomes and X chromosomes is presented in Supplementary Figure S1. The availability of phenotypic records varied substantially across traits and parities. For instance, in the first parity, the number of animals with recorded data ranged from 519 for TLWT_D21 to 1902 for NWEAN (Table 1), reflecting the heterogeneity in data coverage across traits.

**Figure 1:**
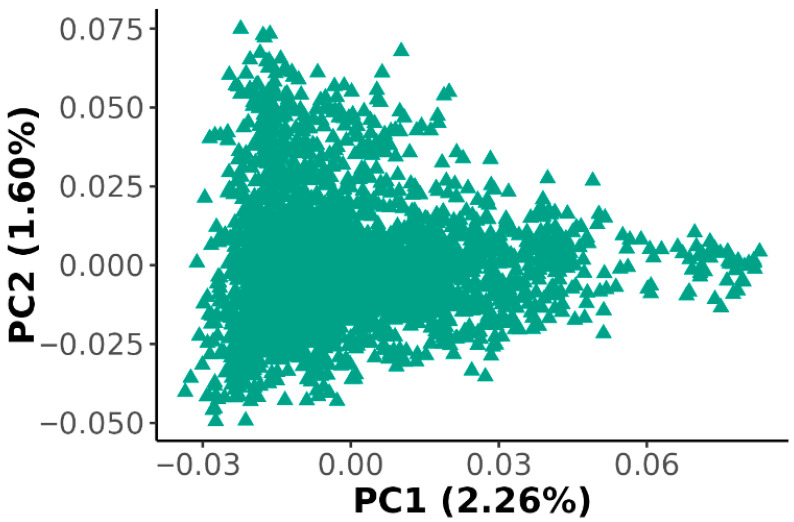
Principal component analysis showing the relationship between the two principal components (PC1, PC2) and the proportion of genetic variance explained (percentage of variation explained) in pigs.

Principal component analysis (PCA) revealed a continuous distribution of individuals along the first two principal components (PC1: 2.26%; PC2: 1.60%), with no clear clustering (Figure 1). This indicates an absence of population stratification within the reference population; hence, principal components were not included as covariates in the correlation or GWAS analyses.

### 3.2. Estimation of Genetic Parameters

Heritability analysis revealed that the majority of genetic variance for most traits was attributable to autosomal SNPs (Table 2). For example, the heritability of TLWT_BA_P3 was largely autosomal ($h_{AUT}^{2}$ = 0.1536 ± 0.0630), with only a modest X chromosome contribution ($h_{X}^{2}$ = 0.0141 ± 0.0250). Overall, total heritability ($h^{2}$) estimates ranged from 0.0369 (TLWT_BA_P1) to 0.2146 (TNB_P3). Partitioning by chromosome showed that autosomal heritability ($h_{AUT}^{2}$) varied between 0.0369 (TLWT_BA_P1) and 0.154 (TLWT_BA_P3); whereas X-linked heritability ($h_{X}^{2}$) ranged from 0 (TLWT_BA_P2) to 0.109 (TNB_P3).

In most traits, the X chromosome contributed less than 5% of the total heritability ${(h}^{2}$ < 5%), although notable exceptions were observed. In the third parity of TNB, the X chromosome accounted for approximately 50.9% of the total heritability ($h_{X}^{2}$ = 0.109 ± 0.050 of $h^{2}$= 0.215 ± 0.062). Similarly, for NWEAN_P2, X-linked variants explained roughly one-quarter of the total heritability ($h_{X}^{2}$ = 0.016 ± 0.017, representing ~21.5% of $h^{2}$ = 0.073 ± 0.040).

Notably, for several traits (TNB_P1–P2, TLWT_BA_P1–P2, and TLWT_D21_P2–P3), the estimated X-chromosomal heritability ($h_{X}^{2}$) was 0. This indicates that the analyses converged successfully, but no detectable X-linked additive genetic variance remained after adjusting for fixed effects and covariates, suggesting that, for these traits, the genetic basis is predominantly influenced by autosomal rather than X-linked loci.

**Figure 2:**
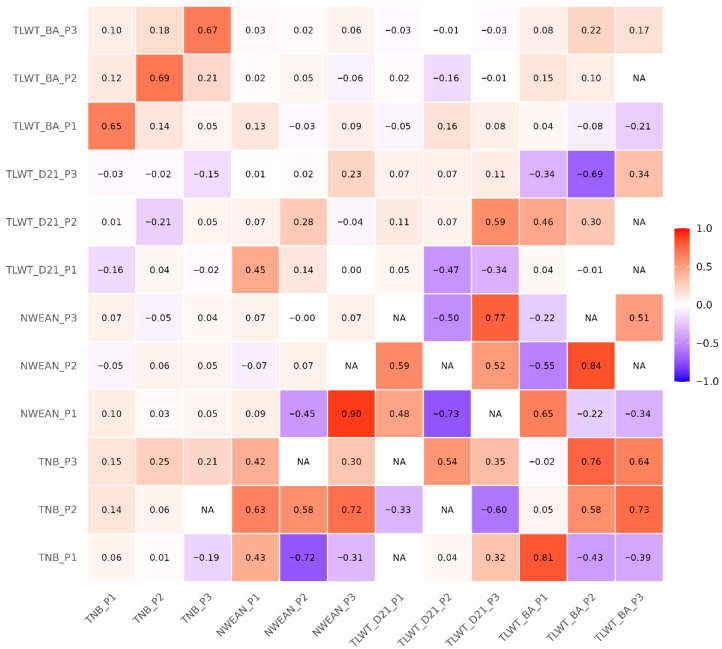
Heritability (diagonal), Genetic (lower triangle), and Phenotypic (upper triangle) correlation coefficient among the first three parities of the four reproductive traits in Yorkshire pigs. In this diagram, positive correlations are depicted using the color blue, while negative correlations are represented by the color red.

Genetic and phenotypic correlations among reproductive traits were generally consistent, indicating a shared genetic basis for reproductive performance across parities (Figure 2). The strongest positive genetic correlation ($r_{G}\;=\;0.90\;\pm \;0.67$) was observed between NWEAN_P1 and NWEAN_P3, suggesting that the number of piglets weaned is strongly influenced by stable genetic factors across reproductive cycles. Moderate-to-strong positive genetic correlations $\left(r_{G}\;=\;0.65\;to\;0.81\right)$ among TLWT_BA_P1, NWEAN_P1, and TNB_P1, suggesting that improved litter birth weight and total number born could concurrently enhance weaning performance. Conversely, negative genetic correlations were identified between TNB_P1 and NWEAN_P2 $\left(r_{G}\;=\;-0.72\;\pm \;0.48\right)$ and between NWEAN_P1 and TLWT_D21_P2 $\left(r_{G}\;=\;-0.73\;\pm \;0.71\right),$ reflecting possible trade-offs between early and later reproductive outcomes.

Phenotypic correlations between TNB and TLWT_D21 were negative ($r_{P}$ ≈ −0.16 to −0.21) between TNB and TLWT_D21, suggesting an inverse relationship between litter size and postnatal litter weight gain. Strong correlations across successive parities, such as TLWT_BA_P2 with TNB_P2 ($r_{P}=0.69$), indicate that, within the same parity, sows producing larger litters tend to have greater total litter weight at birth.

### 3.3. Genome-Wide Association Studies (GWAS)

**Figure 3:**
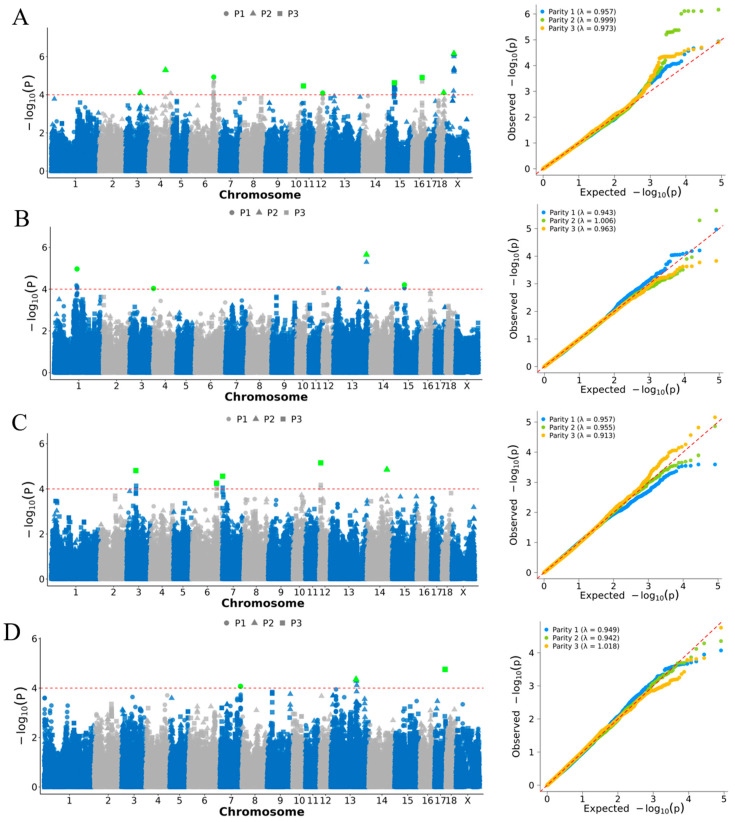
Manhattan and Quantile–Quantile (QQ) plots for genome-wide association analysis of (**A**) NWEAN, (**B**) TLWT_BA, and (**C**) TLWT_D21. (**D**) TNB traits; *x*-axis indicates chromosomes, and *y*-axis indicates −log_10_ (*p*-value). In the Manhattan plots, parity groups are denoted by point shape: P1 = circles (●), P2 = squares (■), and P3 = triangles (▲). Lead SNPs are highlighted in green-filled shapes. The horizontal dashed line on the Manhattan plots represents the suggestive significance threshold ($p<1\times 10^{-4}$), while the diagonal red line in the Q–Q plots represents the expected values under the null hypothesis.

Genome-wide association studies were performed for each of the four reproductive traits across the first three parities to investigate their genetic basis. A total of 56, 12, 11, and 4 suggestively significant SNPs $\left(p<1\times 10^{-4}\right)$ were detected for NWEAN, TLWT_BA, TLWT_D21, and TNB, respectively. Of the SNPs associated with NWEAN, 15 were located on the X chromosome, whereas no suggestively significant SNPs were detected on the X chromosome for the other traits. The genomic inflation factor (λ = 0.94 on average) indicated effective control of population stratification and other confounding factors (e.g., breed effects), as reflected by the QQ plots (Figure 3). After conditional analysis, 10, 5, 5, and 3 independent GWAS signals were identified for NWEAN, TLWT_BA, TLWT_D21, and TNB, respectively (Figure 3).

**Figure 4:**
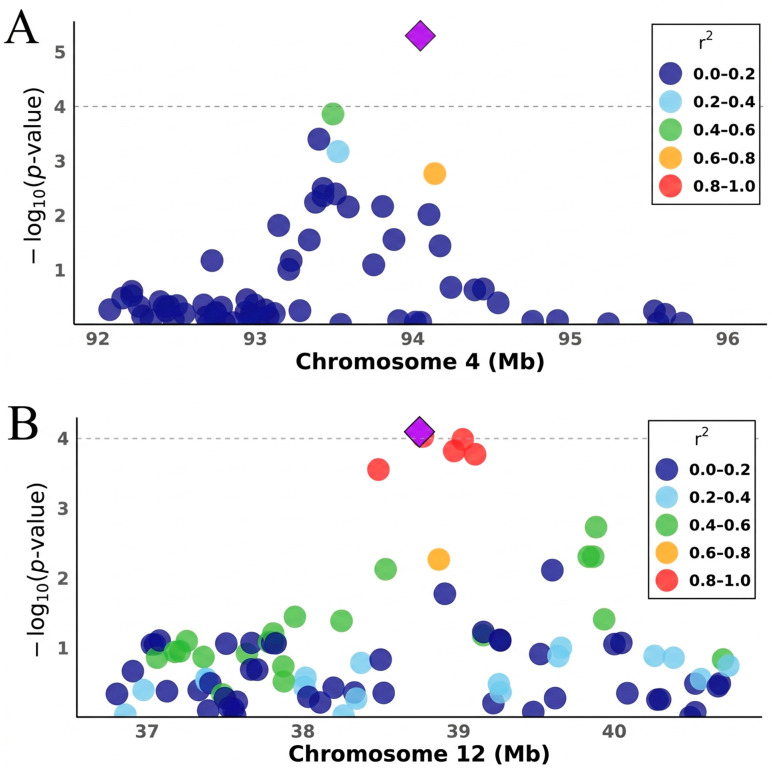

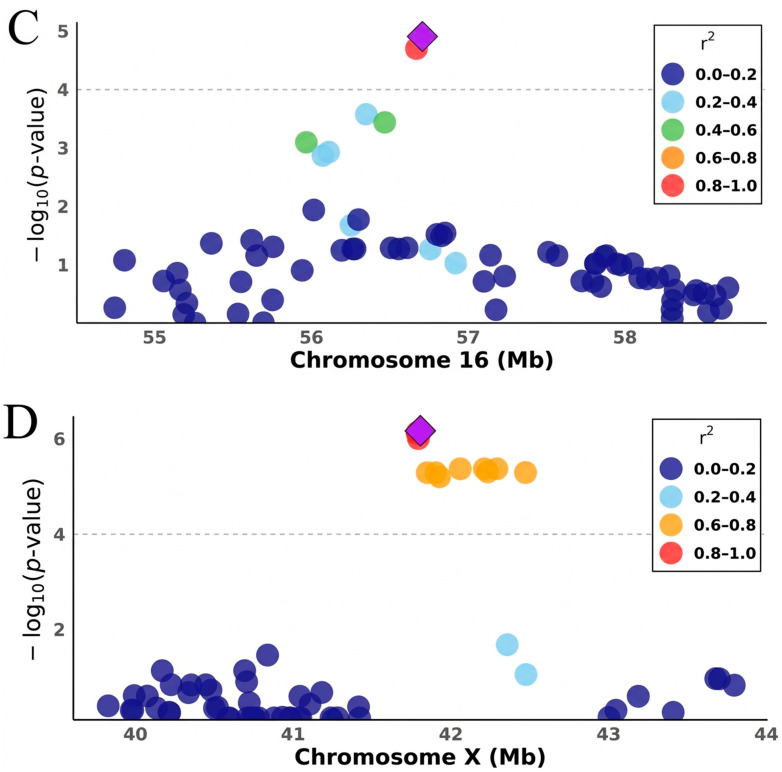
Regional association plots illustrating NWEAN associations across three parities. (**A**) *ARHGEF2* on chromosome 4 in the second parity; (**B**) *ACACA* on chromosome 12 in the first parity; (**C**) *TENM2* on chromosome 16 in the third parity; and (**D**) *RBM10* on chromosome X in the second parity. The lead SNP is indicated by a purple diamond. Surrounding variants are shown as circles and are colored according to linkage disequilibrium with the lead SNP ($r^{2}$), where pink denotes strong LD ($r^{2}\;=\;0.8\;-\;1.0$). Each plot highlights association signals and local linkage disequilibrium.

Several candidate genes, including *ARHGEF2*, *TENM2*, *ACACA*, and *RBM10*, were identified within a ±1 Mb window of independent lead signals associated with NWEAN, the latter of which is located on the X chromosome (Figure 4). TLWT_BA-associated gene included *GOLM2*, located on chromosome 1 in parity 1 (P1). For TLWT_D21, candidate genes, such as *SLC25A28* and *SLC44A5*, were identified on chromosomes 14 (P2) and 6 (P3), respectively. Candidate genes associated with TNB included *KCNK13* and *ADCY5*, located on chromosomes 7 (P1) and 13 (P2), respectively. No suggestively significant SNPs or independent SNPs were detected for TLWT_BA_P3 or TLWT_D21_P1. Details of all significant SNPs and corresponding candidate genes are summarized in Table 3.

### 3.4. Functional Enrichment Analysis

**Figure 5:**
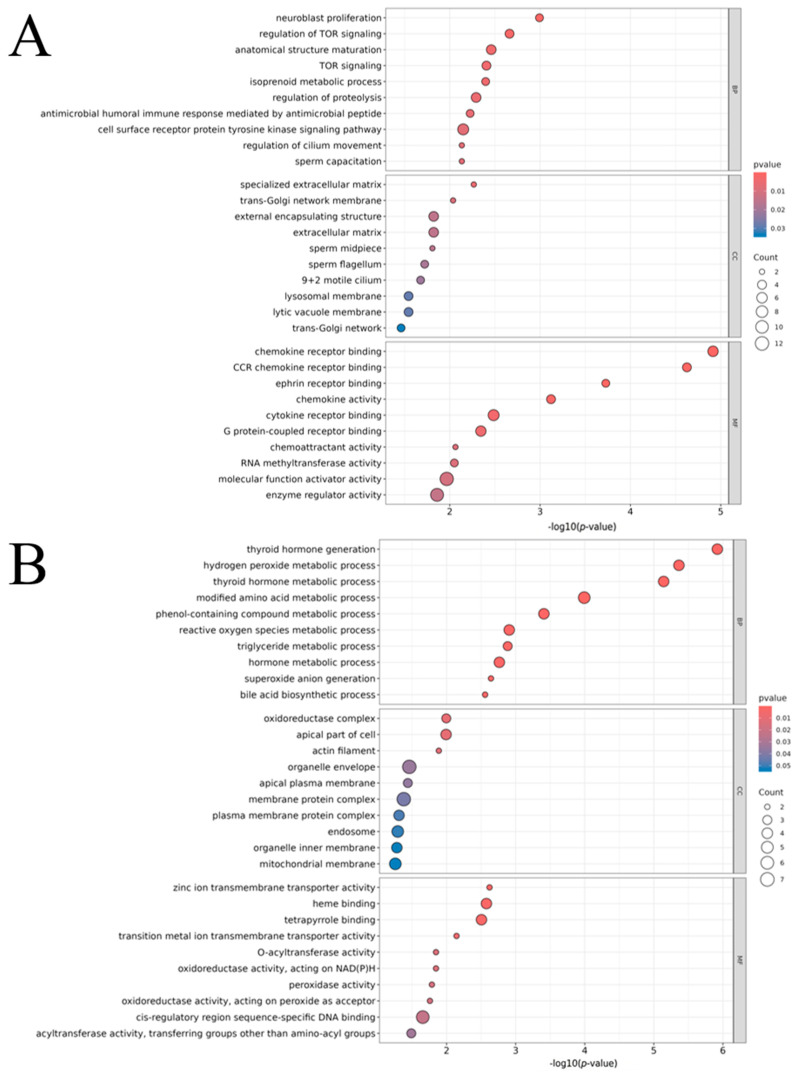

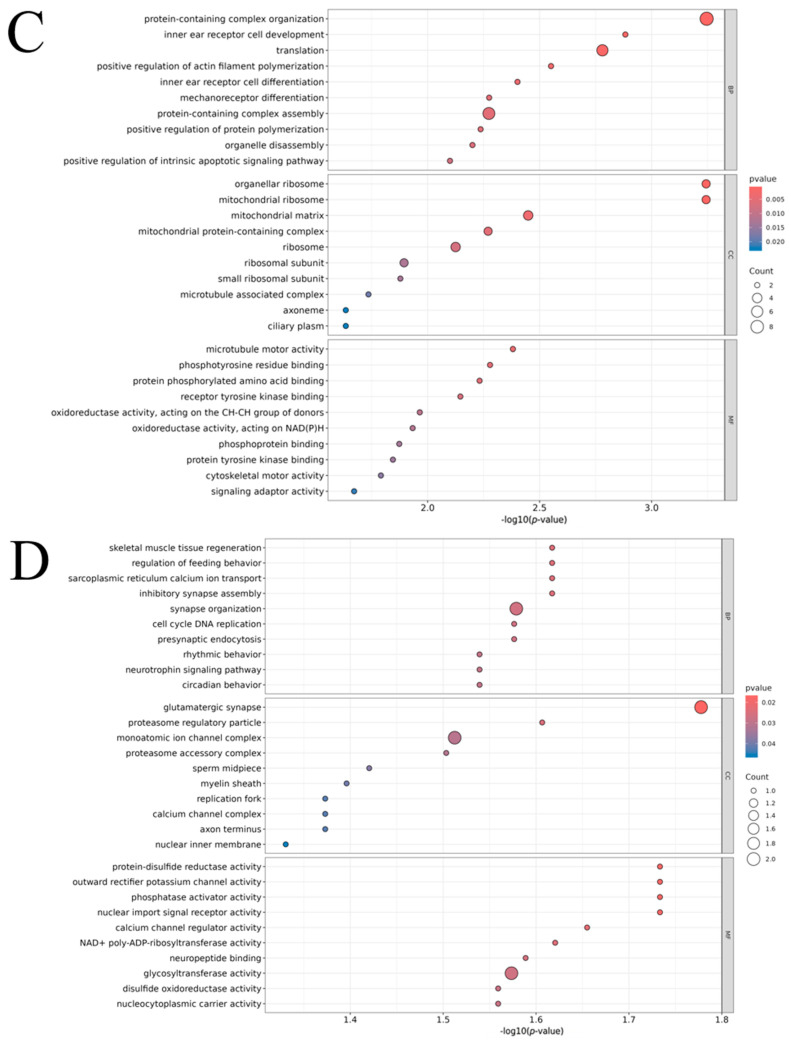
GO enrichment analysis of significant SNPs for the four reproductive traits with combined parities, highlighting the top 10 terms per ontology category for (**A**) NWEAN, (**B**) TLWT_BA, (**C**) TLWT_D21, and (**D**) TNB.

**Figure 6:**
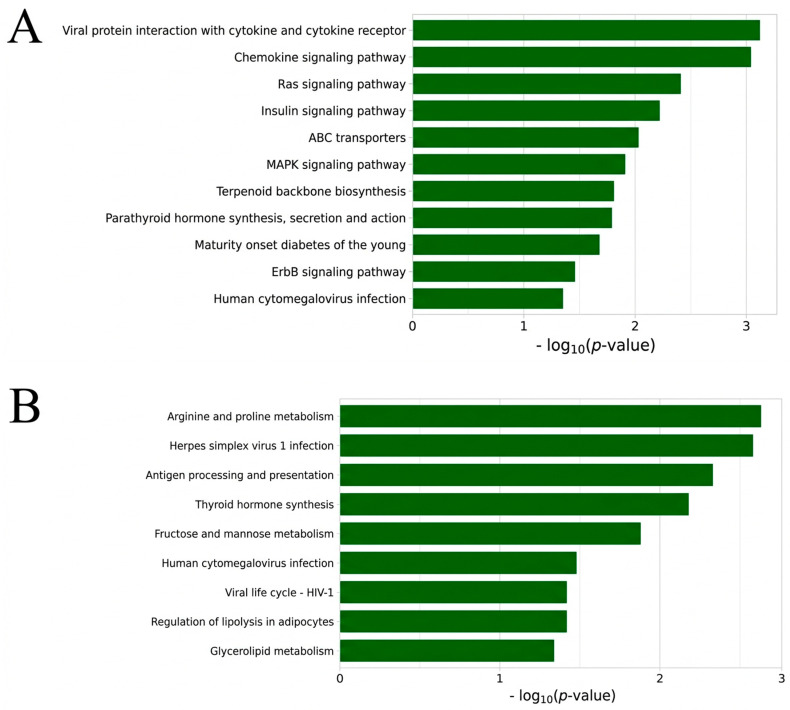

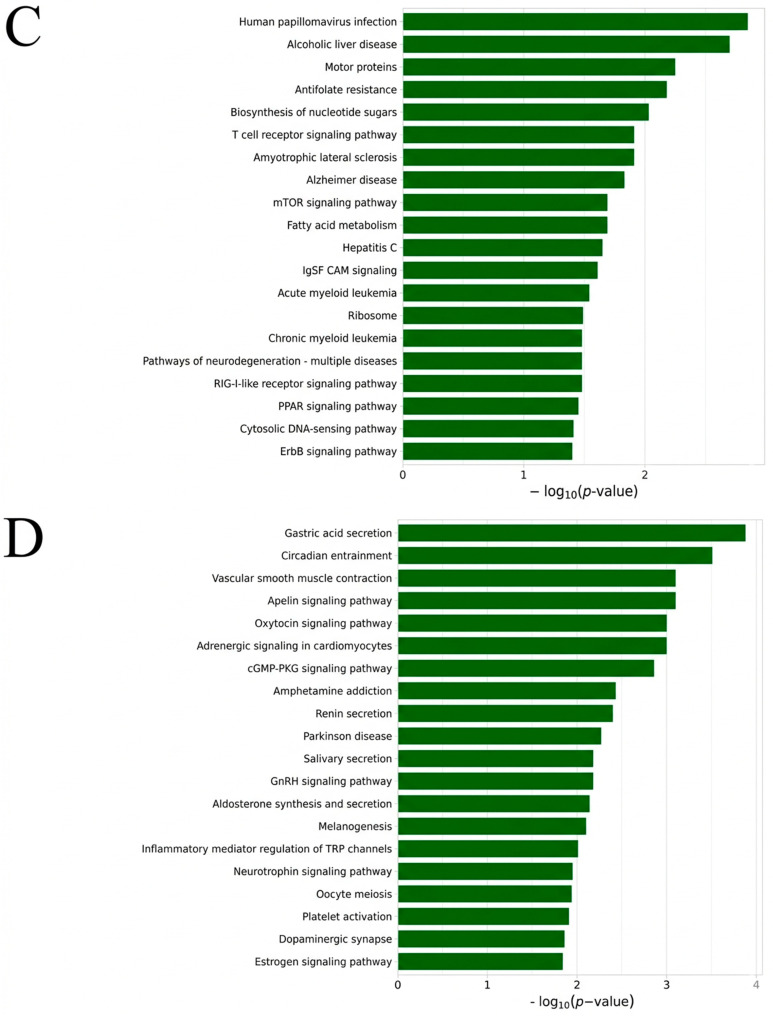
KEGG pathway enrichment analysis of significant SNPs for the combined parities of the four reproductive traits: (**A**) NWEAN, (**B**) TLWT_BA, (**C**) TLWT_D21, and (**D**) TNB.

Enrichment analysis of positional candidate genes (±1 Mb from lead SNPs) revealed clear, trait-specific functional patterns (Figure 5 and Figure 6). For NWEAN, GO terms were predominantly related to sperm structure and motility, including the sperm midpiece, sperm flagellum, and 9 + 2 motile cilium. This points to a central role for cilia and flagella biology, with genes such as *DNAI2* and *SPACA5* contributing to these signals (Figure 5A). In contrast, TLWT_BA showed enrichment for endocrine and metabolic processes, including thyroid hormone generation and metabolism, as well as reactive oxygen species-related pathways, suggesting that variation influencing early body weight is more closely tied to systemic metabolic regulation (Figure 5B). TLWT_D21 showed enrichment for processes involved in protein complex organization and translation, along with mitochondrial and ribosomal components and cytoskeletal features, indicating that cellular growth, energy production, and protein synthesis may shape weaning weight at day 21 (Figure 5C). In addition, TNB was characterized by enrichment of neuroendocrine-related functions, including synapse organization, calcium handling, and rhythmic or circadian-associated processes, which is consistent with hormonal and neural regulation of reproductive output (Figure 5D).

KEGG pathway analysis supported these trait-level differences (Figure 6). Both NWEAN and TLWT_BA showed enrichment for growth and metabolic signaling pathways, including insulin signaling and *ErbB*, *Ras*, and *MAPK*-related signaling, involving genes such as *ACACA*, *ARAF*, *ELK1*, and *SHC1* (Figure 6A,B). TLWT_D21 highlighted pathways that reflect broader cellular regulation and several disease-linked signaling modules (Figure 6C). In contrast, TNB was enriched for circadian entrainment and multiple hormone and neuroactive signaling pathways, including GnRH, oxytocin, estrogen signaling, and dopaminergic synapse pathways, further supporting a neuroendocrine contribution to litter size regulation (Figure 6D). Notably, several highlighted candidates, including *SPACA5*, *ELK1*, and *ARAF*, are located on the X chromosome, suggesting that X-linked variation may contribute to these regulatory networks.

## 4. Discussion

Understanding the genetic basis of reproductive traits is crucial for enhancing pig breeding efficiency. While previous studies have primarily focused on autosomal chromosomes, the role of X chromosomes has often been overlooked. This study aims to address this by examining the genetic basis of key reproductive traits (TLWT_BA, TNB, TLWT_D21, and NWEAN) in Yorkshire pigs through integrating information from both autosomes and the X chromosome. The inclusion of the X chromosome is particularly significant due to its potential for sex-specific effects and its substantial gene content related to reproduction, as it may harbor genes affecting sex-biased inheritance and maternal productivity.

The contribution of the X chromosome to heritability varied across traits and parities, ranging from 0 to 0.109, indicating that the X chromosome may exert a notable influence on the genetic basis of key reproductive traits in sows. Later-parity traits showed higher heritability than early-parity traits, which is consistent with previous reports on pedigree-based models [32] and genomic relationship matrices (G-matrix or GBLUP-type approaches) [33] showing increasing heritability with parity [5]. The X chromosome accounted for approximately half of the total heritability of TNB in the third parity and roughly one-quarter of the total heritability of NWEAN in the second parity, indicating a substantial influence of the X chromosome in these traits. These results align with studies in cattle [24,34], sheep [35], and humans [36], emphasizing that ignoring the X chromosome can lead to underestimated genetic contribution. Overall, incorporating both autosomal and X-linked effects in genomic selection models is critical for accurate breeding value prediction and balanced selection strategies, thereby optimizing reproductive performance in breeding programs.

Candidate genes such as *ARHGEF2*, *RBM10*, and *ACACA* were noteworthy for NWEAN, with functional evidence for these genes coming mainly from mammalian studies. Specifically, *ACACA* has been implicated in humans [37], while studies in livestock species, including pigs [38] and cattle [39], support roles for these candidates in economically important traits. *ACACA*, in particular, is known to influence reproduction by regulating lipid synthesis, which is essential for oocyte maturation and embryo development. Recent studies have demonstrated that *ACACA* plays a major role in lipid metabolism [37] and follicular growth [40], and it has been associated with the insulin signaling pathway. The insulin signaling pathway is pivotal in regulating follicular development and mediating metabolic signals that influence reproductive hormone synthesis and action, consistent with previous studies on reproductive genetics in dairy cattle [41,42].

The identification of *RBM10* as an X-linked locus represents a novel finding in pigs, as most GWAS have focused on autosomal regions [43]. *RBM10* is known to play key roles in RNA binding and embryogenesis [39]. Moreover, X chromosome-associated genes such as *ARAF* and *ELK1* in NWEAN were linked to the insulin signaling [44] and ErbB signaling pathways, both of which have sex-specific roles in gametogenesis and hormone regulation [45]. The ErbB signaling pathway was also associated with genes such as *GRB2* and *NCK2* in TLWT_D21.

Additionally, *ARAF* and *ARHGEF2* were associated with the regulation of proteolysis, a process crucial for ovarian follicular development and ovulation that involves the remodeling of the extracellular matrix and tissue structures [41]. These results highlight the specialized role of the X chromosome in reproductive and fertility traits, consistent with findings in cattle [34]. Although X-linked genes represent a smaller fraction of the genome, they exert disproportionately large effects on reproduction due to their unique inheritance patterns and sex-biased influences [46,47].

*ADCY5* emerged as a strong candidate gene for TNB, as it was associated with multiple KEGG pathways, including the cGMP–PKG signaling pathway. The enrichment in this pathway suggests a functional role in calcium-dependent smooth muscle contraction and ovarian steroidogenesis, underscoring its importance in the regulation of ovulation and uterine activity [48]. Previous studies have reported diverse roles of *ADCY5* across species, including seasonal estrus in sheep [49], egg production in ducks [50], fertility in cattle [51], and associations with gestational duration, fetal development, and birth weight in humans [52,53]. However, its function in pig reproduction has not been thoroughly investigated.

*SLC44A5* was identified as a key regulator of fetal growth, likely through its role in choline transport, linking it to birth outcomes and reproductive efficiency in livestock [54]. The enrichment of motor protein pathways highlights the significance of ciliary- and microtubule-based movement in gamete transport and embryo development [55]. *DGAT1* and *B2M* were associated with glycerolipid metabolism and antigen processing pathways in TLWT_BA, respectively, highlighting the interplay between maternal energy balance, lipid mobilization, and immune competence in sustaining fertility and piglet survival [56,57]. Moreover, *DGAT1* was also associated with triglyceride metabolism, which is directly linked to energy supply, a critical factor for successful folliculogenesis and embryo development [41].

Genes, such as *DNAI2*, *NHERF1*, *DEFB1*, and *SPACA5*, were identified for their potential roles in sperm motility [58] and ciliary function [59], through GO terms such as sperm flagellum and 9 + 2 motile cilium. The GO term sperm flagellum highlights the significance of genes in the formation and function of this structure, which is essential for overall sperm motility and morphology. Similarly, *CALM1* and *DEFB1* were both enriched in the GO term sperm midpiece, indicating their potential roles in sperm structure and energy metabolism [60]. Furthermore, *ARHGEF2* was enriched in the GO term positive regulation of developmental process, highlighting its contributions to reproductive and developmental regulation [61]. Overall, the results show that pig fertility depends on endocrine, metabolic, and immune pathways that can serve as targets for genetic improvement.

Despite uncovering novel loci for reproductive traits, several limitations should be noted. The sample size and statistical power may have restricted the detection of small-effect variants, while marker density and coverage were limited, as imputation was not performed. Consistent with the limited sample size, estimates of additive genetic correlation also exhibited relatively large standard errors, reducing confidence in the precision of those correlation estimates. Additionally, the female-only dataset introduces sex-specific bias, and X chromosome inactivation (XCI) may create genetic architectures that differ from autosomes, even in female-only cohorts, which could affect effect size estimation. Future studies incorporating larger and sex-balanced cohorts, as well as allele-specific expression or epigenetic data, would allow the application and comparison of multiple X chromosome-specific analytical strategies, such as XCI-aware models or Bayesian frameworks, potentially improving the detection of X-linked loci and refining genetic correlation estimates. Nevertheless, this study provides a foundation for further exploration of candidate genes identified through GWAS on both autosomes and X chromosomes, offering valuable insights into the genetic basis of reproductive traits in Yorkshire pigs.

## 5. Conclusions

This study demonstrates that both autosomal and X-chromosomal loci contribute to reproductive traits in Yorkshire pigs, with the X chromosome exerting a notable, trait-specific influence on litter size and number weaned. Genome-wide association analyses identified key candidate genes, including *ARHGEF2*, *ACACA*, *SPACA5*, and *ELK1*, which are involved in sperm structure, follicular development, lipid metabolism, and reproductive signaling pathways. Several X-linked genes suggest potential sex-specific regulatory effects on fertility. These findings highlight the importance of incorporating X-chromosomal variation into genomic selection programs to improve reproductive efficiency. Overall, this work provides new insights into the genetic basis of sow fertility and identifies actionable targets for enhancing breeding strategies.

## Figures and Tables

**Table 1:** Descriptive statistics for phenotypic values of reproductive traits recorded in Yorkshire.

Trait	N	Min	Max	Median	Mean	SD	CV (%)
TNB_P1	2129	2	23	13	12.53	2.94	23.48
TNB_P2	1017	2	26	14	13.76	3.37	24.49
TNB_P3	558	5	24	14	14.25	3.22	22.59
NWEAN_P1	1902	1	30	10	10.12	1.76	17.34
NWEAN_P2	899	3	30	11	10.87	1.94	17.83
NWEAN_P3	524	2	30	11	11.02	2.27	20.64
TLWT_BA_P1 (kg)	2103	1.2	26	12	11.84	3.46	29.23
TLWT_BA_P2 (kg)	991	1.2	29	15	14.97	3.94	26.33
TLWT_BA_P3 (kg)	534	3	25.6	15.85	15.71	3.55	22.59
TLWT_D21_P1 (kg)	519	26.04	106.28	70.4	69.27	11.27	16.26
TLWT_D21_P2 (kg)	631	15.16	156.04	68.7	70.08	11.30	16.13
TLWT_D21_P3 (kg)	432	17.2	138.18	74.79	74.37	13.55	18.22

TLWT_BA total litter weight of piglets born alive (kg), TNB total number born, TLWT_D21 total litter weight at 21 days (kg), and NWEAN number of weaned pigs (individual).

**Table 2:** Overall heritability ($h^{2}$) and heritability due to autosomes ($h_{AUT}^{2}$) and X chromosome ($h_{X}^{2}$).

Trait	$h^{2}$	$h_{\_SE}^{2}$	$h_{AUT}^{2}$	$h_{AUT\_SE}^{2}$	$h_{X}^{2}$	$h_{X\_SE}^{2}$
TNB_P1	0.0558	0.0205	0.0558	0.0200 **	0	0.0048
TNB_P2	0.0594	0.0345	0.0594	0.0335 *	0	0.0088
TNB_P3	0.2146	0.0620	0.1054	0.0486 *	0.1093	0.0502 *
NWEAN_P1	0.0911	0.0269	0.0886	0.0266 ***	0.0025	0.0052
NWEAN_P2	0.0726	0.0404	0.0570	0.0378	0.0156	0.0167
NWEAN_P3	0.0657	0.0631	0.0641	0.0621	0.0016	0.0127
TLWT_BA_P1	0.0370	0.0190	0.0370	0.0186 *	0	0.0041
TLWT_BA_P2	0.0964	0.0401	0.0964	0.0394 **	0	0.0081
TLWT_BA_P3	0.1676	0.0655	0.1536	0.0630 **	0.0141	0.0250
TLWT_D21_P1	0.0544	0.0579	0.0537	0.0563	0.0007	0.0145
TLWT_D21_P2	0.0714	0.0527	0.0714	0.0508	0	0.0150
TLWT_D21_P3	0.1057	0.0758	0.1057	0.0721	0	0.0263

TLWT_BA total litter weight of piglets born alive (kg), TNB total number born, TLWT_D21 total litter weight at 21 days (kg), and NWEAN number of weaned pigs (individual); ***, **, and *, significant at *p* < 0.001, *p* < 0.01, and *p* < 0.05, respectively.

**Table 3:** Independently associated SNPs and genes in which they are located were identified in the genome-wide association study after conditional analysis of the reproductive traits.

Trait	SNP	Chr	Position	*p* Value	Nearest Gene
TLWT_BA_P1	1_127142034	1	127,142,034	$1.08\times 10^{-5}$	GOLM2
TLWT_BA_P1	4_19906	4	19,906	$9.26\times 10^{-5}$	
TLWT_BA_P1	13_27151296	13	27,151,296	$8.95\times 10^{-5}$	
TLWT_BA_P1	15_47809293	15	47,809,293	$6.22\times 10^{-5}$	
TNB_P1	7_111896622	7	111,896,622	$8.50\times 10^{-5}$	KCNK13
NWEAN_P1	6_134445578	6	134,445,578	$1.17\times 10^{-5}$	ADGRL4
NWEAN_P1	12_38748038	12	38,748,038	$8.08\times 10^{-5}$	ACACA
TLWT_BA_P2	13_184055780	13	184,055,780	$2.22\times 10^{-6}$	
TNB_P2	13_137134779	13	137,134,779	$4.43\times 10^{-5}$	ADCY5
TLWT_D21_P2	14_110809250	14	110,809,250	$1.38\times 10^{-5}$	SLC25A28
NWEAN_P2	3_82335316	3	82,335,316	$7.73\times 10^{-5}$	
NWEAN_P2	4_94047783	4	94,047,783	$5.03\times 10^{-6}$	ARHGEF2
NWEAN_P2	4_123756546	4	123,756,546	$8.40\times 10^{-5}$	FNBP1L
NWEAN_P2	18_38843367	18	38,843,367	$7.77\times 10^{-5}$	DPY19L1
NWEAN_P2	23_41805827	X	41,805,827	$6.80\times 10^{-7}$	RBM10
TNB_P3	17_56217737	17	56,217,737	$1.75\times 10^{-5}$	
TLWT_D21_P3	3_50114620	3	50,114,620	$1.53\times 10^{-5}$	
TLWT_D21_P3	6_137812334	6	137,812,334	$5.55\times 10^{-5}$	SLC44A5
TLWT_D21_P3	7_1400566	7	1,400,566	$2.72\times 10^{-5}$	
TLWT_D21_P3	12_6629321	12	6,629,321	$6.96\times 10^{-6}$	
NWEAN_P3	11_7803554	11	7,803,554	$3.44\times 10^{-5}$	B3GLCT
NWEAN_P3	15_37230998	15	37,230,998	$2.36\times 10^{-5}$	
NWEAN_P3	16_56704434	16	56,704,434	$1.24\times 10^{-5}$	TENM2

## Data Availability

All genotypic and phenotypic data used in this analysis are available from the corresponding author upon reasonable request.

## Supplementary Materials

The following supporting information can be downloaded at: https://www.mdpi.com/article/10.3390/ani16050750/s1, Figure S1: Distribution of genetic markers across all autosomes and the X chromosome in the Large White pig population. This figure illustrates marker density and genomic coverage used for downstream analyses, ensuring adequate representation of both autosomal and sex chromosomes. Figure S2: Correlation between autosomal and X chromosome genomic relationship matrix (GRM) values. This analysis evaluates the consistency and genetic relatedness captured across autosomal and X-linked markers within the population. Table S1: Pairwise correlations between traits, including phenotypic correlation and genetic correlation, together with their standard errors. Table S2: Detailed information on all candidate genes identified from the GWAS analyses, including genomic locations, associated traits, and functional annotations. Table S3: Comparison of identified loci with previously reported pig GWAS signals (QTLdb overlap) to assess locus novelty. Table S4: Phenotypic variance explained (PVE%) by each identified locus.

## Abbreviations

The following abbreviations are used in this manuscript:

$h^{2}$	Heritability
$h_{AUT}^{2}$	Heritability of autosomes
$h_{X}^{2}$	Heritability of X chromosome
$r_{G}$	Genetic Correlations
$r_{P}$	Phenotypic Correlations
GO	Gene Ontology
GRM	Genomic Relationship Matrix
GWAS	Genome-wide association studies
KEGG	Kyoto Encyclopedia of Genes and Genomes
LD	Linkage Disequilibrium
MAF	Minor Allele Frequency
Mb	Megabase
MLMA	Mixed Linear Model Association
NWEAN	Number of Weaned pigs
PCA	Principal Component Analysis
PCs	Principal Components
SNPs	Single Nucleotide Polymorphisms
TLWT_BA	Total Litter Weight of piglets born alive
TLWT_D21	Total Litter Weight at 21 days
TNB	Total Number Born
