# Supplementary material for: Genome-Wide Association Studies on the Autosomes and Chromosome X Uncover Genetic Basis of Reproductive Traits in Yorkshire Pigs
Source: Animals (Basel). 2026 Feb 27;16(5):750. doi: 10.3390/ani16050750 (PMC12984400; doi:10.3390/ani16050750)
Supplement: Supplementary file 1 [file animals-16-00750-s001.zip › Supplementary Figure S2.pdf]

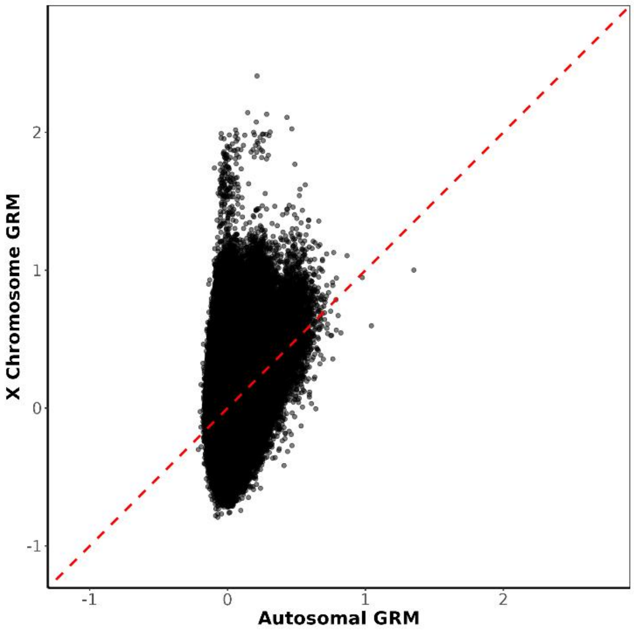

Figure. S2. Correlation between autosomal and X-chromosome genomic relationship matrix (GRM) values
