# Supplementary material for: Genome-Wide Association Studies on the Autosomes and Chromosome X Uncover Genetic Basis of Reproductive Traits in Yorkshire Pigs
Source: Animals (Basel). 2026 Feb 27;16(5):750. doi: 10.3390/ani16050750 (PMC12984400; doi:10.3390/ani16050750)
Supplement: Supplementary file 1 [file animals-16-00750-s001.zip › Supplementary Table S4.pdf]

Supplementary Table S4. Phenotypic variance explained (PVE%) by each identified locus

Trait	SNP	Chr	Position	*p Value*	PVE%
			First Parity		
TLWT_BA	1_127142034	1	127142034	1.08×10^{-5}	0.7841
TLWT_BA	4_19906	4	19906	9.26×10^{-5}	0.6948
TLWT_BA	13_27151296	13	27151296	8.95×10^{-5}	0.3094
TLWT_BA	15_47809293	15	47809293	6.22×10^{-5}	0.3603
TNB	7_111896622	7	111896622	8.50×10^{-5}	0.1610
NWEAN	6_134445578	6	134445578	1.17×10^{-5}	0.8092
NWEAN	12_38748038	12	38748038	8.08×10^{-5}	0.7821
			Second Parity		
TLWT_BA	13_184055780	13	184055780	2.22×10^{-6}	0.1094
TNB	13_137134779	13	137134779	4.43×10^{-5}	0.7229
TLWT_D21	14_110809250	14	110809250	1.38×10^{-5}	2.4232
NWEAN	3_82335316	3	82335316	7.73×10^{-5}	1.7596
NWEAN	4_94047783	4	94047783	5.03×10^{-6}	2.3884
NWEAN	4_123756546	4	123756546	8.40×10^{-5}	1.4514
NWEAN	18_38843367	18	38843367	7.77×10^{-5}	1.5344
NWEAN	23_41805827	X	41805827	6.80×10^{-7}	2.6648
			Third Parity		
TNB	17_56217737	17	56217737	1.75×10^{-5}	1.7377
TLWT_D21	3_50114620	3	50114620	1.53×10^{-5}	4.1942
TLWT_D21	6_137812334	6	137812334	5.55×10^{-5}	1.8101
TLWT_D21	7_1400566	7	1400566	2.72×10^{-5}	4.3567
TLWT_D21	12_6629321	12	6629321	6.96×10^{-6}	2.2516
NWEAN	11_7803554	11	7803554	3.44×10^{-5}	2.8788
NWEAN	15_37230998	15	37230998	2.36×10^{-5}	3.3314
NWEAN	16_56704434	16	56704434	1.24×10^{-5}	2.5937
