# Supplementary figures and images for: Genome-Wide Association Studies on the Autosomes and Chromosome X Uncover Genetic Basis of Reproductive Traits in Yorkshire Pigs
Source: Animals (Basel). 2026 Feb 27;16(5):750. doi: 10.3390/ani16050750 (PMC12984400; doi:10.3390/ani16050750)

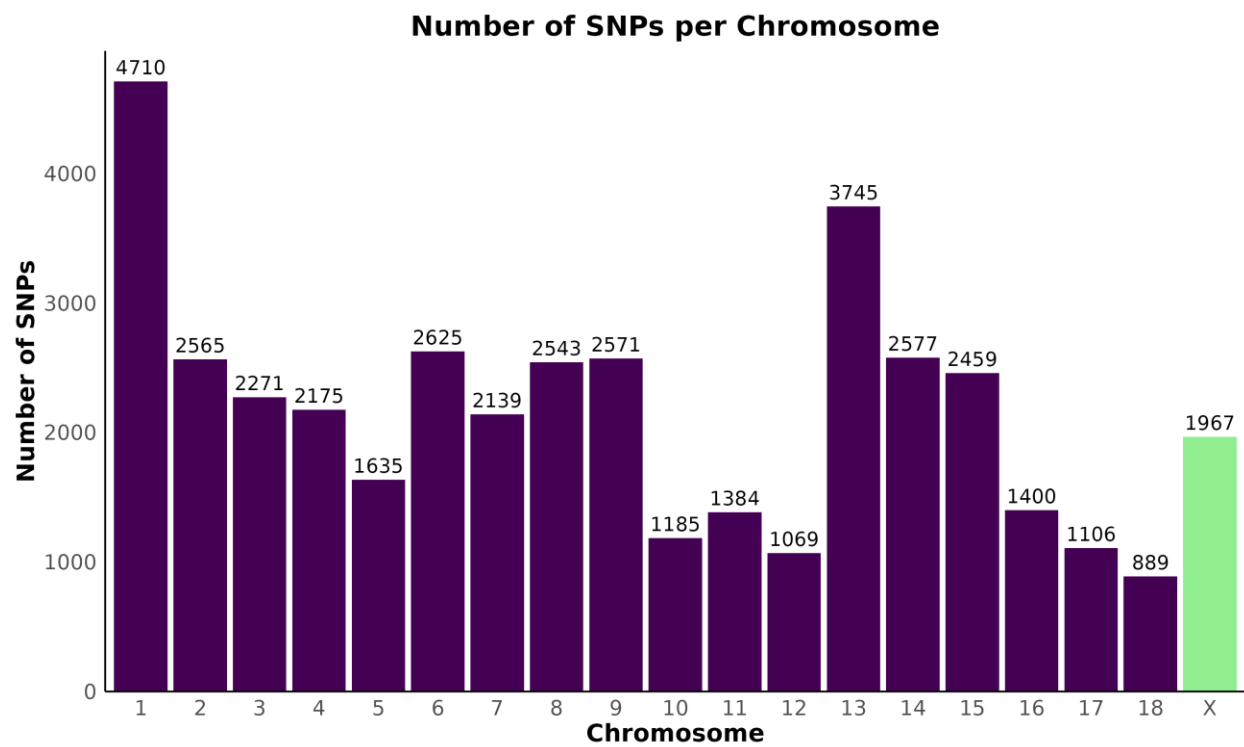

Figure. S1: Distribution of markers on both autosomes and X-chromosomes.

Supplement: Supplementary file 1 [file animals-16-00750-s001.zip › Supplementary Figure S1.pdf]
